# Supplementary material for: Compressing deep graph convolution network with multi-staged knowledge distillation
Source: PLoS One. 2021 Aug 13;16(8):e0256187. doi: 10.1371/journal.pone.0256187 (PMC8363007; doi:10.1371/journal.pone.0256187)
Supplement: Attachment — Submitted filename: rebuttal_letter.pdf [file pone.0256187.s001.pdf]

Compressing Deep Graph Convolution Network with Multi-Staged Knowledge Distillation

Junghun Kim, Jinhong Jung, and U Kang

We would like to thank the reviewers for their high quality reviews and constructive comments. Below, we summarize how we addressed the reviewers' requirements.

1. Reviewer 1.
   - **(R1-1)** Does the proposed model has the ability to gain the full knowledge about all the features when there are multiple layers?
     - **(A1-1)** Accuracy of MustaD improves as the number of GCN layers in the teacher increases. This implies that MustaD gains more knowledge from the given input features when more GCN layers are used in the teacher. In other words, MustaD has the ability to gain the full knowledge about all the features when there are multiple layers in the teacher. We reported this in lines 334-336 in experiments section.
   - **(R1-2)** Does the author considers the semantic problems of features in the proposed method?
     - **(A1-2)** Since the main purpose of MustaD is to compress a deep GCN model to a compact model while preserving the multi-hop feature aggregation of the deep model, it is hard to expect that MustaD considers the semantic problems of features. We added the discussion on this as a future work in conclusion section (lines 447-448).
   - **(R1-3)** What happens to the Knowledge Distillation when the teachers accuracy is too low? Does the proposed system helps in solving these issues?
     - **(A1-3)** Through the experiments summarized in Table 3, we have shown that MustaD has an ability to aggregate information from farther nodes than the teacher, thus relieving the student's strong dependence on the performance of the teacher. We reported this in lines 341-345 in experiments section.
   - **(R1-4)** In the process of KD, does the training errors in teacher propagate to student directly? Can the proposed system identify the errors?
     - **(A1-4)** Fig 4 shows that MustaD compensates for the training error of merely propagating the prediction of the teacher. We discussed this in lines 420-425 in experiments section.
   - **(R1-5)** Please cite the following papers: 1) Ashokkumar P, Siva Shankar G, Gautam Srivastava, Praveen Kumar Reddy Maddikunta, and Thippa Reddy Gadekallu. 2021. A Two-stage Text Feature Selection Algorithm for Improving Text Classification. ACM Trans. Asian Low-Resour. Lang. Inf. Process. 20, 3, Article 49 (April 2021), 19 pages. DOI:https://doi.org/10.1145/3425781, and 2) G. Siva Shankar, P. Ashokkumar, R. Vinayakumar, Uttam Ghosh, Wathiq Mansoor, Waleed S. Alnumay, "An Embedded-Based Weighted Feature Selection Algorithm for Classifying Web Document", Wireless Communications and Mobile Computing, vol. 2020, Article ID 8879054, 10 pages, 2020. https://doi.org/10.1155/2020/8879054
     - **(A1-5)** We added the two references (lines 65-67 in related work section).
2. Reviewer 2.
   - **(R2-1)** The quality of the figures can be improved more. Figures should be eye-catching. It will enhance the interest of the reader.
     - **(A2-1)** We improved Figures 1 and 2. In particular, we redrew Fig 1 to be more informative, which previously looked complicated, and added markups for Fig 2 to be eye-catching.
   - **(R2-2)** The abstract is long and NOT satisfactory. It should contain the following parts: 1) The importance of or motivation for the research, 2) The issue/argument of the research, 3) The methodology, 4) The result/findings, and 5) The implications of the result/findings.
     - **(A2-2)** We rewrote the abstract to contain those five parts.
   - **(R2-3)** Please highlight the contribution clearly in the introduction.
     - **(A2-3)** We revised introduction section to emphasize the contribution of MustaD (lines

32-43 in introduction section). Furthermore, the contents of the contribution list have also been revised to clearly highlight the contributions (lines 54-56 in introduction section).

- **(R2-4)** In the first four paragraphs of literature review section, the authors have presented a good references, but they need to present the recent and most updated references.
  - **(A2-4)** We added four up-to-date references (lines 65-67 in related work section).
- **(R2-5)** In the literature review section, you need to be consistent in the use of the verb tense, it is common to use the past tenses.
  - **(A2-5)** We reviewed the literature review section to be consistent in the use of the verb tense.
- **(R2-6)** The summary at the end of the literature review should be focused on the limitations of related work.
  - **(A2-6)** We added the limitations of deep GCNs at the end of the literature review (lines 104-106 in related work section).
- **(R2-7)** The discussion is very important in research paper. Nevertheless, this section is short and should be presented completely.
  - **(A2-7)** We included more complete discussion (lines 334-336, lines 341-345, lines 380-387, lines 420-425, and lines 430-433 in experiments section).
- **(R2-8)** Major contribution was not clearly mentioned in the conclusion part.
  - **(A2-8)** We revised the conclusion part to clearly present our major contribution.
- **(R2-9)** Make sure the Conclusion succinctly summarizes the paper. It should not repeat phrases from the Introduction!
  - **(A2-9)** We revised conclusion section so that the conclusion succinctly summarizes the paper.
- **(R2-10)** Authors should add the most recent reference: 1) CANintelliIDS: Detecting In-Vehicle Intrusion Attacks on a Controller Area Network using CNN and Attention-based GRU, and 2) DeepAMD: Detection and identification of Android malware using high-efficient Deep Artificial Neural Network, Future Generation Computer Systems 115, 844-856.
  - **(A2-10)** We added the two references (lines 65-67 in related work section).
